# Enhancing scanning electron microscopy imaging quality of weakly conductive samples through unsupervised learning

**DOI:** 10.1038/s41598-024-57056-4

**Published:** 2024-03-18

**Authors:** Xin Gao, Tao Huang, Ping Tang, Jianglei Di, Liyun Zhong, Weina Zhang

**Affiliations:** Key Laboratory of Photonic Technology for Integrated Sensing and Communication, Ministry of Education, Guangdong University of Technology, Guangzhou, 510006 China

**Keywords:** Materials science, Nanoscale materials, Computational science

## Abstract

Scanning electron microscopy (SEM) is a crucial tool for analyzing submicron-scale structures. However, the attainment of high-quality SEM images is contingent upon the high conductivity of the material due to constraints imposed by its imaging principles. For weakly conductive materials or structures induced by intrinsic properties or organic doping, the SEM imaging quality is significantly compromised, thereby impeding the accuracy of subsequent structure-related analyses. Moreover, the unavailability of paired high–low quality images in this context renders the supervised-based image processing methods ineffective in addressing this challenge. Here, an unsupervised method based on Cycle-consistent Generative Adversarial Network (CycleGAN) was proposed to enhance the quality of SEM images for weakly conductive samples. The unsupervised model can perform end-to-end learning using unpaired blurred and clear SEM images from weakly and well-conductive samples, respectively. To address the requirements of material structure analysis, an edge loss function was further introduced to recover finer details in the network-generated images. Various quantitative evaluations substantiate the efficacy of the proposed method in SEM image quality improvement with better performance than the traditional methods. Our framework broadens the application of artificial intelligence in materials analysis, holding significant implications in fields such as materials science and image restoration.

## Introduction

Due to its exceptional sub-nanometer resolution and large depth of field, scanning electron microscopy (SEM) has emerged as a crucial tool for morphological characterization at the submicron scale^1^, and it is widely used in materials science^2^, biomedicine^3^, chemistry^4^ and so on. SEM generates high-resolution images by focusing an electron beam onto the sample surface and detecting the emitted secondary electrons. Therefore, a prerequisite for obtaining high-resolution and high-quality SEM images is the high conductivity of samples^5^. Weakly conductive samples, such as most polymers and some semiconductor materials, often exhibit an excess of electrons or free charges on their surface, which impedes the transmission of electronic signals, resulting in a significant reduction in imaging contrast and clarity. Besides, organic contamination introduced during material synthesis or processing can also diminish the electrical conductivity of the samples^6,7^. Subject to the electron beam, the organic matter decomposes into carbon-hydrogen compounds and covers the sample surface. This will lead to the charge accumulation and thus diminish the quality of SEM imaging^8,9^. To improve the sample conductivity, a common method is coating the sample surface with a gold film through vacuum sputtering^10^. However, the gold layer will cover the original material information, resulting in a reduction in elemental contrast for the material. For samples at the scale of hundreds of nanometers or even smaller, the gold layer will obscure the details of the sample structure, leading to a misrepresentation of surface structures. Additionally, gold-coated samples are generally non-reusable, increasing experimental costs and operational complexities. Therefore, there is an urgent need for a method that can quickly, conveniently, and effectively improve the SEM imaging quality of weakly conductive samples without contaminating or damaging the sample.

With the advancement of computational imaging, image post-processing provides another avenue for enhancing SEM imaging quality. Traditional methods such as linear^11^ or nonlinear filters^12,13^ recover sharp images from blurred images by deconvolution methods. They are mostly achieved by simplifying and modeling the principles of blurring, and then utilizing prior information from the images to restore the blurred images. However, in practical situations, the types of blurring are more complex than those modeled. At the same time, iterative calculation of the blur kernel requires a significant amount of time. Compared to traditional methods, neural networks can automatically learn the blur kernel without the need for manual design, and they exhibit faster computational speeds^14–20^. Therefore, deep learning-based methods have been widely applied to enhance micrograph quality, such as image deblurring^21–24^ and super-resolution^25–29^. For SEM images, Haan et al.^30^ used a Generative Adversarial Network (GAN) to increase the resolution of SEM images by two fold. Juwon et al. proposed a multi-scale network for deblurring defocused SEM images, achieving superior performance compared to traditional methods^31^. Although deep learning has achieved significant advancements in SEM imaging improvement, the existing studies primarily rely on supervised learning, which requires paired data containing both blurred and clean images for network training. However, in practical scenarios involving weakly conductive samples, it is challenging to obtain one-to-one corresponding SEM images with both blurred and clear versions under the same field of view. Hence, there is a pressing need for an unsupervised learning approach that can perform image deblurring without relying on paired data training.

In recent years, the characteristics of Cycle-consistent Generative Adversarial Network (CycleGAN) unpaired training make unsupervised learning possible^32^, and demonstrate comparable performance to supervised methods. This framework has been successfully applied to enhance the quality of natural^33,34^, satellite^35^, and fluorescence microscopic images^36–38^. Here, we propose an unsupervised learning-based approach to improve the quality of SEM images captured from weakly conductive samples. The proposed method employs the CycleGAN architecture to learn from unpaired data consisting of blurry and clear SEM images in an end-to-end manner. An additional edge loss function was introduced into the CycleGAN model to address the requirements of material structure analysis, helping eliminate artifacts and restore detailed information about the material contours. Multiple image evaluation metrics demonstrated that the improved CycleGAN model can effectively enhance the SEM image quality of various weakly conductive samples without any complicated physical operations.

## Principle and network analysis

**Figure 1:**
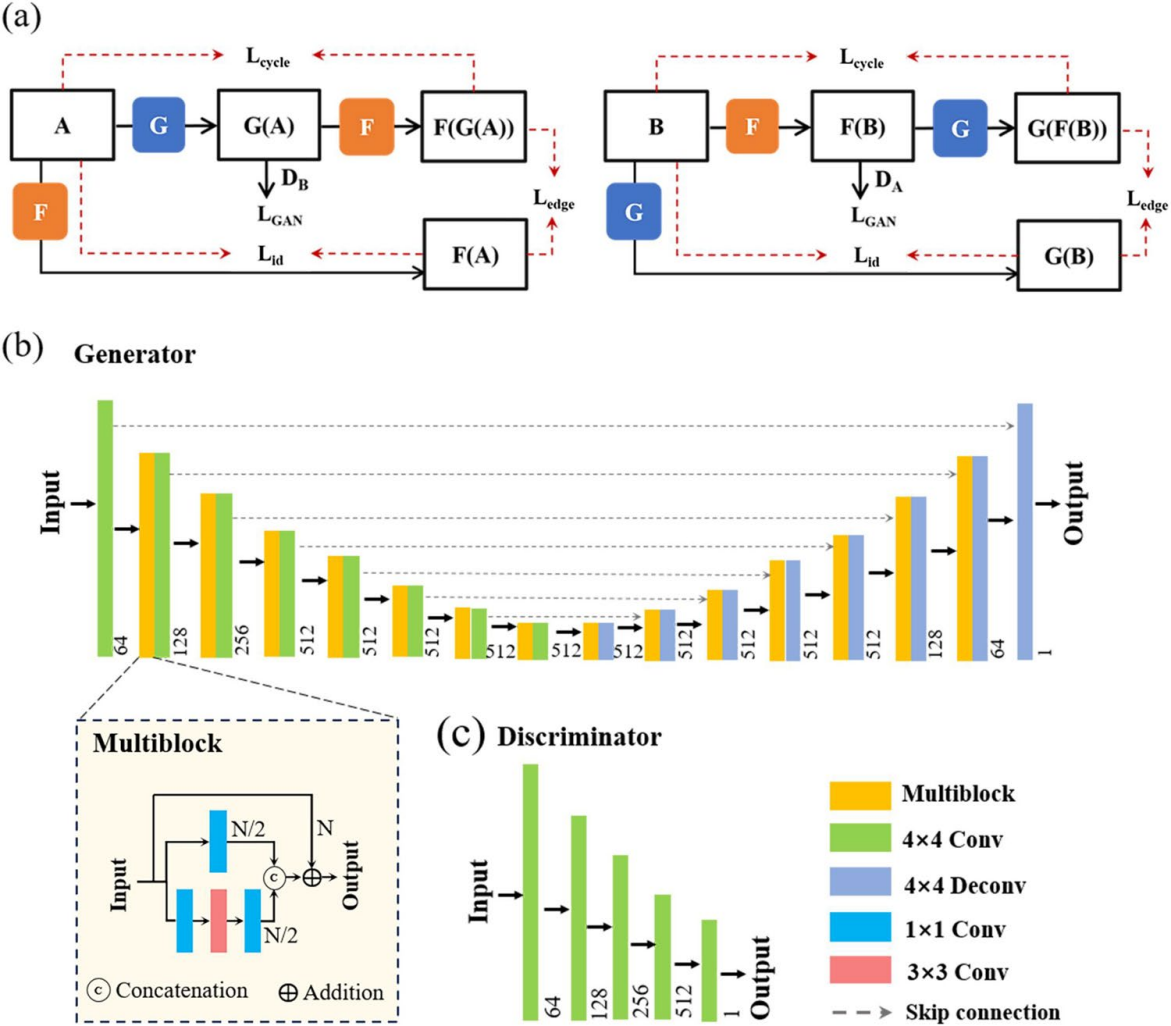
(**a**) Schematic of the overall architecture. The proposed method consists of two generators (*G* and *F*) and two discriminators ($D_{A}$ and $D_{B}$). The generator *G* predicts clean images from blurred image *A*, Discriminator $D_A$ attempts to distinguish between the real clear image and the generated clear image. The generator *F* predicts a blurred image from clean image *A*, and the discriminator $D_{B}$ attempts to distinguish between the real blurred image and the generated blurred image. Loss functions include adversarial loss ($L_{GAN}$), cyclic consistency loss ($L_{cycle}$), identity loss ($L_{id}$), and edge loss ($L_{edge}$). (**b**) The generator network structure. Numbers below each layer represent the number of channels. (**c**) The discriminator network structure.

The overall framework of our method is shown in Fig. 1a, which is inspired by CycleGAN. It consists of two generators (*G* and *F*) and two discriminators ($D_{A}$ and $D_{B}$). *A* and *B* represent the blurred and clear image sets, respectively, and no pre-aligned image pairs are required in the two image collections. Generator *G* aims to translate the blurred image *A* to a clear one *G*(*A*). The discriminator $D_{B}$ determines whether *G*(*A*) is a real or generated clear image. Generator *F* aims to translate the clear image *B* to a blurred one *F*(*B*). The discriminator $D_A$ determines whether *F*(*B*) is a real or generated blurred image. These generators and discriminators are trained using adversarial loss ($L_{GAN}$), which allows the generator to complete the conversion between different image domains. To address the gradient vanishing problem and generate high-quality images, the $L_{GAN}$ employed least squares loss instead of cross-entropy loss. The cycle-consistency loss ($L_{GAN}$) is imposed to make the cycle-generated images as close to the input images as possible. Here, the Structure Similarity Index Measure (SSIM)^39^ loss is used as the $L_{cycle}$, which can measure the similarity between the initial input images *A* and *B* and the corresponding cyclic images *F*(*G*(*A*)) and *G*(*F*(*B*)) output by two generators in terms of brightness, contrast, and structure. The utilization of $L_{GAN}$ and $L_{cycle}$ allow the network to be trained with unpaired data. In addition, blurred image *A* and clear image *B* are input into generators *F* and *G* to construct identity loss ($L_{id}$) and edge loss ($L_{edge}$), respectively. The $L_{id}$ is used to ensure that the information from the original input image is retained. The $L_{edge}$ utilizes the Sobel operator to extract image edge information and preserves the edge detail information of the image. This is necessary because just using the weak constraint introduced by cycle consistency is prone to generate noise artifacts and structural distortion in the output images when our datasets consist of SEM images of various materials with different morphologies. The equations for the loss functions can be seen in Method. The generators *G* and *F* are trained simultaneously to learn the mapping relationship between the two image domains.

**Figure 2:**
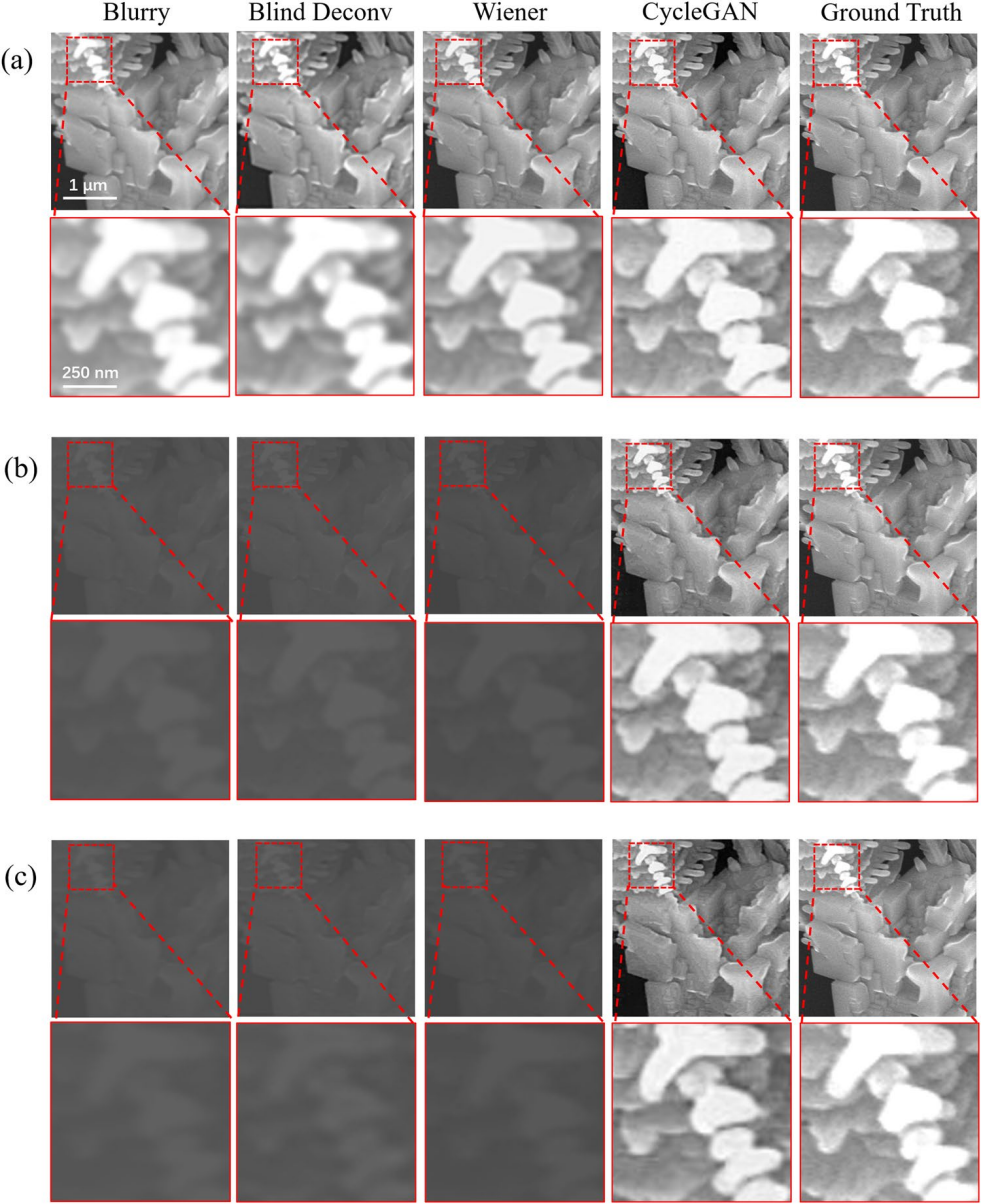
Deblurring results of different models on simulated datasets. The material in the SEM images is iron chloride. (**a**) Deblurring results for data with Gaussian blur only. (**b**) Deblurring results for data with Gaussian blur and synthetic fog. (**c**) Deblurring results for data with synthetic fog and hybrid blur (Gaussian blur, motion blur, out-of-focus blur).

**Figure 3:**
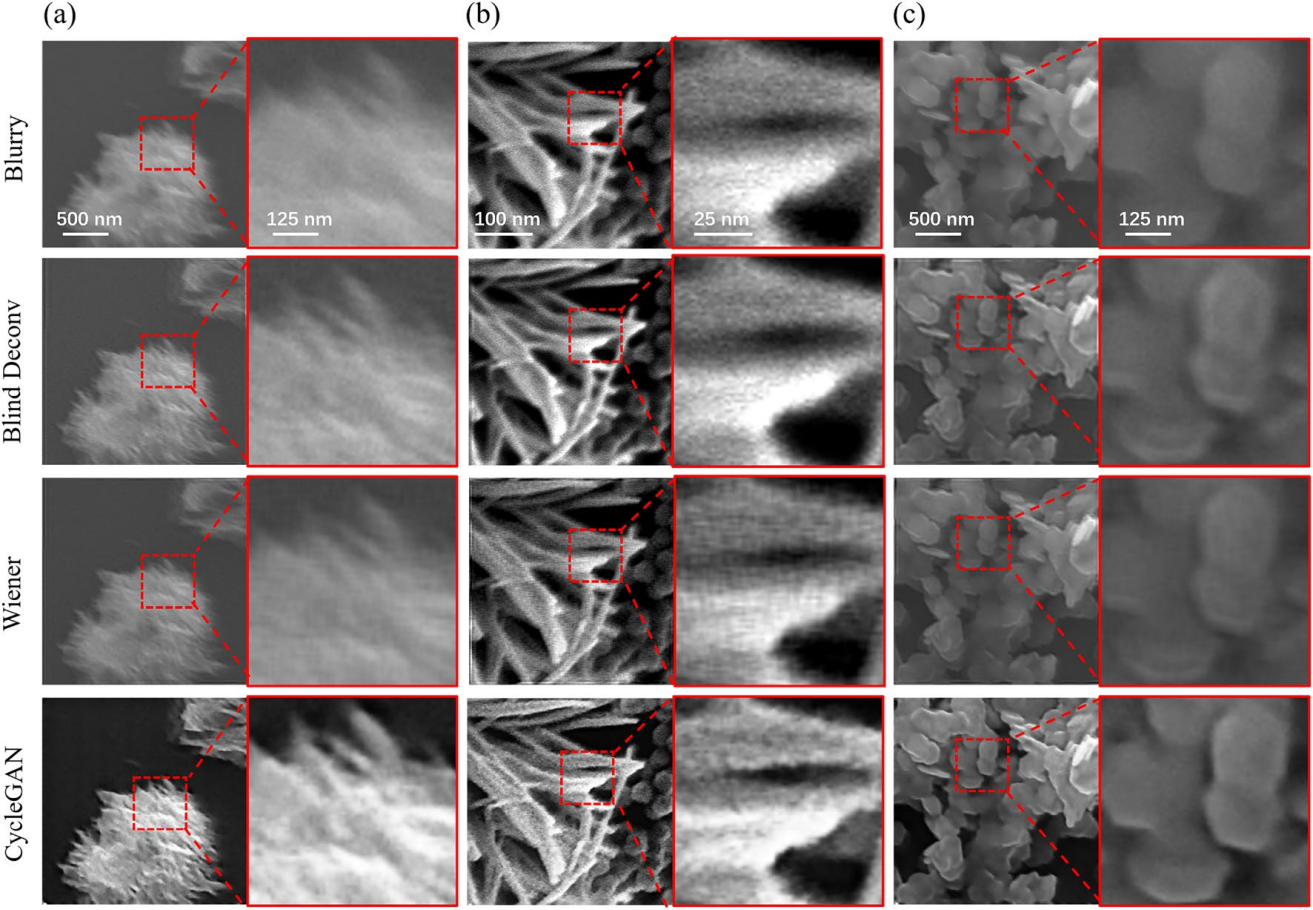
Deblurring results of different models on real datasets. The materials in SEM images were tungsten trioxide (${\text{WO}}_{3}$) (**a**, **c**) and copper sulfide (CuS) (**b**).

Figure 1b and c show the structure of the generator and discriminator, respectively. We designed a Unet network structure with multi-scale convolution as our generator, which was inspired by inception blocks^21^ and Unet^40^. The designed generator has 8 convolution layers and 8 deconvolution layers. Each convolution layer is followed by an instance norm and an activation function (leaky ReLU). Except for the stride size of the eighth convolution layer and the first deconvolution layer is 1, the other convolution stride sizes are 2. In addition, there are 14 Multi blocks, whose structure is shown in the inset in Fig. 1b. Multi block can enhance image edge features by using multi-scale convolution, to better recover image details. Each Multi block consists of 1$\times$1 convolution kernels and 3$\times$3 convolution kernels, and all convolution stride sizes are 1. Skip connections are used in the middle to fuse information at different scales. The discriminator shown in Fig. 1c was implemented in a full convolution manner. 5 convolution layers were used in the discriminator. Except for the last convolutional layer, each convolution layer was followed by an instance norm and an activation function (leaky ReLU). Except the stride size of the first three convolution layers is 2, the other convolution stride sizes are 1.

## Verification and analysis of experimental results

### Results on the simulated dataset

It is impossible to quantitatively characterize the performance of the model in image enhancement without paired samples. Here, to quantitatively evaluate the effectiveness of the proposed model, the simulated dataset was created comprising pairs of blurred and clear images. Clean SEM images were selected as ground truth and the corresponding low-quality SEM images were synthesized by introducing blur. In response to the weak intrinsic conductivity of the material and the scenario of organic compound doping, three simulated blurry datasets A, B, C were constructed by applying Gaussian blur, Gaussian blur and synthetic fog, hybrid blur (including Gaussian blur, motion blur, out-of-focus blur) and synthetic fog on the clear SEM images, respectively. Figure 2a–c is obtained separately from these three simulated datasets. The kernel size and standard deviation $\sigma$ of the Gaussian blur were set as 7$\times$7 and 1, respectively(detailed information seen in Methods). $\sigma$ = 1 is the level of blurriness that typically occurs in practice. And in practical applications, pixels beyond approximately 3$\sigma$ distance can be considered negligible for the calculation results. Hence, image processing programs only need to compute a (6$\sigma$+1)$\times$(6$\sigma$+1) matrix to ensure the relevant pixel influence. The matrix is the Gaussian blur kernel, whose size was set as 7$\times$7 in our work. The synthetic fog refers to fogging an image to reduce its quality. The degree of fogging is random at different positions in the image(detailed information seen in Methods). Blurry and clear datasets were randomly shuffled to achieve unpaired data training. For comparison, the CycleGAN and the traditional methods such as blind deconvolution (Blind Deconv for short)^41^ and Wiener filtering algorithm (Wiener for short)^42^, were applied to enhance the quality of the simulated blurred images. 10 iterations were set for blind deconvolution. The results are shown in Fig. 2. It can be seen that, for all types of blurry images, CycleGAN demonstrates superior image restoration performance, improving the clarity and contrast of images to approach the ground truth. In contrast, traditional methods such as blind deconvolution and Wiener filtering show poorer performance in handling images with unknown blurry kernels, and it is difficult to recover the contrast and clarity of blurry images that have been modified with added synthetic fog and Gaussian blur.

**Figure 4:**
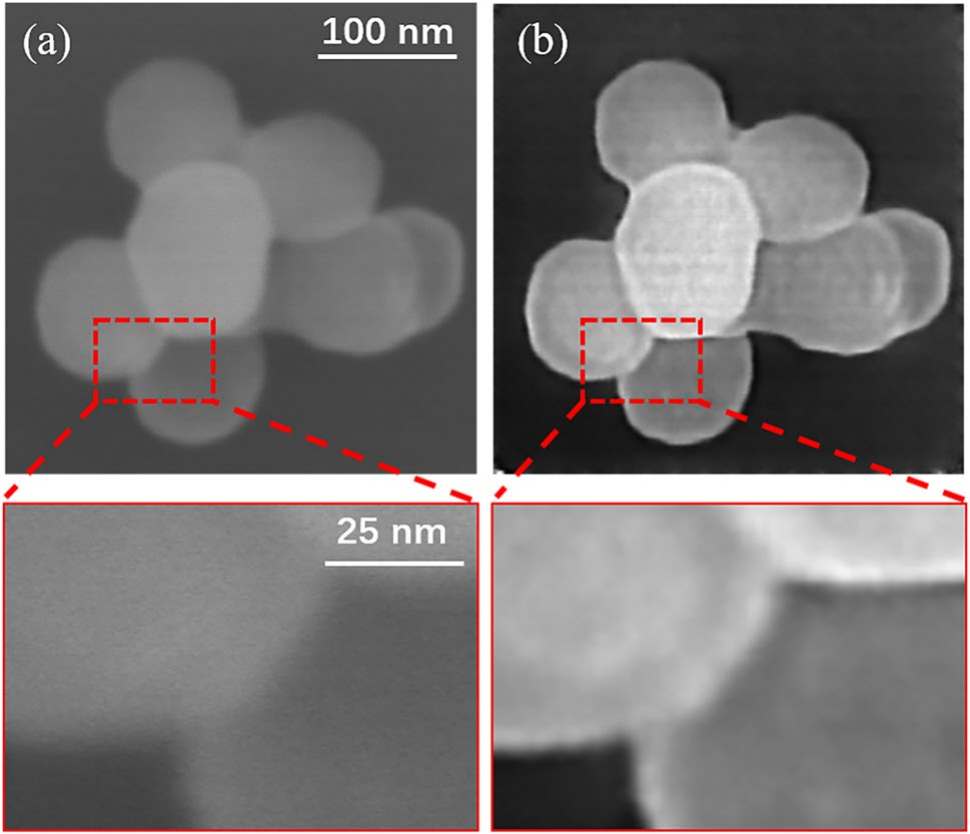
Deblurring results on $\mathrm SiO_2$ SEM images. (**a**) Original SEM image. (**b**) Recovered image by the CycleGAN.

**Figure 5:**
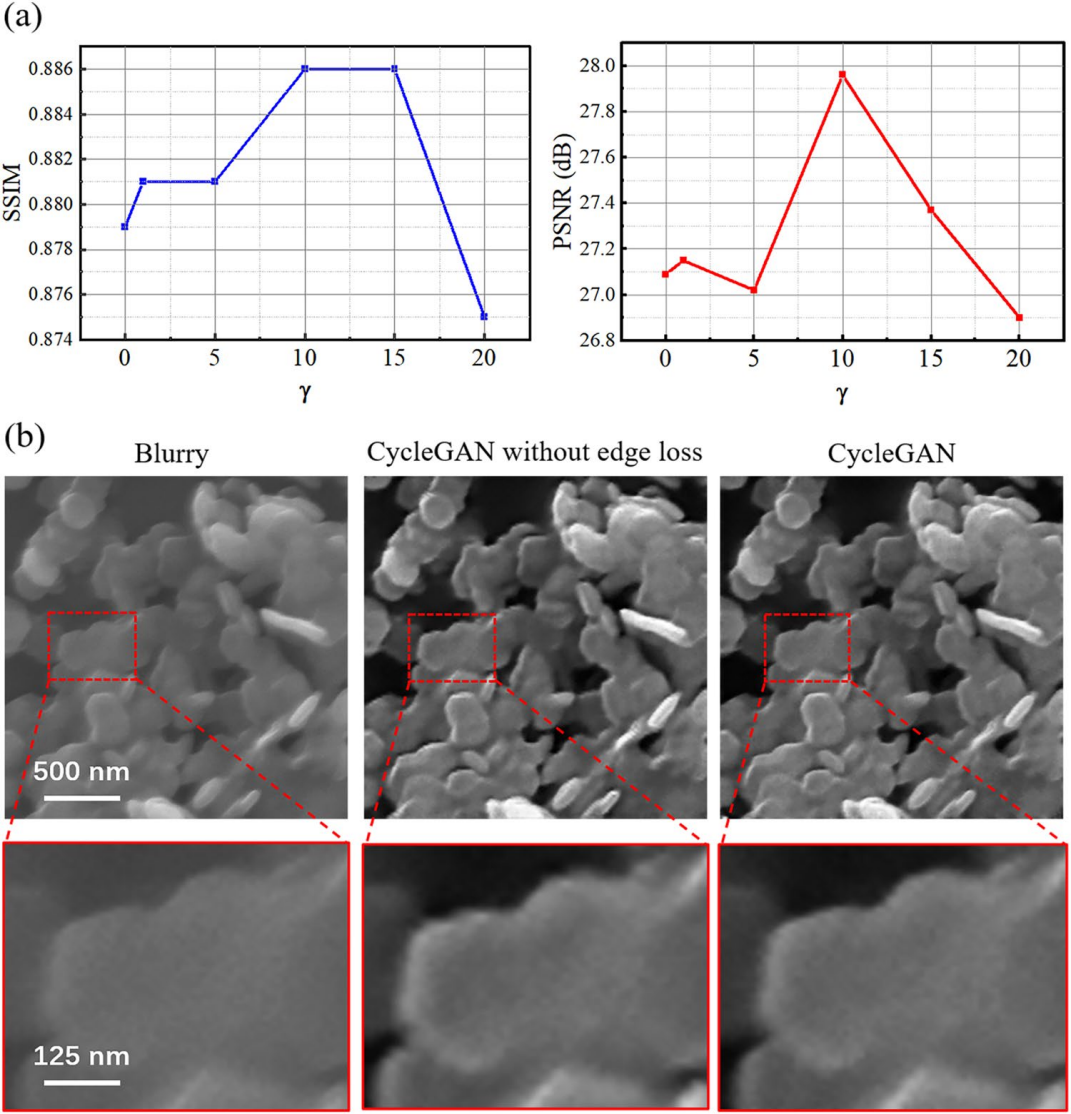
(**a**) Quantitative comparison results of different $\gamma$ values on synthetic data. (**b**) Deblurring results on real data by CycleGAN with and without edge loss. The materials in SEM images are CuS.

To quantitatively evaluate the deblurring results, SSIM^39^ and Peak Signal-to-Noise Ratio (PSNR)^43^ metrics are employed and the average values on the test datasets are shown in Table 1. SSIM measures the image structure similarity by comparing the brightness and contrast between the two images. PSNR is the ratio of the maximum power of the image signal to the noise power (detailed equations seen in Methods). The value range of SSIM is between 0 and 1, where 1 indicates perfect similarity between two images, 0 indicates no similarity. The value range of PSNR is between 0 and infinity, where higher values indicate better image quality. The results show that our method achieves higher SSIM and PSNR scores relative to the traditional methods, especially in datasets B and C, indicating the effectiveness of CycleGAN in improving the SEM imaging quality of weakly conductive samples. To further demonstrate the superiority of the proposed CycleGAN, two other traditional methods, the Richardson–Lucy (RL) algorithm^44^ and constrained least squares (CLS) filter algorithm^45^, have been added for comparison and the results are shown in Supplementary Fig. S1 and Table S1. It can be seen that the performance of the proposed CycleGAN surpasses traditional methods significantly.

**Table 1:** Average SSIM and PSNR of the simulated datasets, and the best results are shown in bold.

Dataset	Metrics	Methods			
		Blurry	Blind Deconv	Wiener	CycleGAN
A	SSIM	0.906	0.901	0.918	**0.929**
	PSNR (dB)	26.97	27.52	27.92	**29.77**
B	SSIM	0.861	0.796	0.845	**0.886**
	PSNR (dB)	25.99	21.73	24.36	**27.96**
C	SSIM	0.771	0.793	0.765	**0.798**
	PSNR (dB)	22.07	21.58	22.24	**24.27**

A represents the dataset that only adds Gaussian blur. B represents the dataset that adds Gaussian blur and the synthetic fog. C represents the dataset that adds hybrid blur (Gaussian blur, motion blur, out-of-focus blur) and the synthetic fog.

**Table 2:** No-reference evaluation indexes values performed on Fig. 2.

Image	Metrics	Methods				
		Blurry	Blind Deconv	Wiener	CycleGAN	Ground truth
a	AG	3.69	4.34	4.40	**6.58**	7.37
	CON	30.69	42.09	44.53	**91.95**	98.59
	SF	7.78	8.91	9.29	**13.47**	13.92
b	AG	0.41	0.79	0.52	**6.28**	7.37
	CON	0.45	1.07	0.64	**83.70**	98.59
	SF	0.94	1.45	1.12	**12.85**	13.92
c	AG	0.35	0.59	0.41	**6.30**	7.37
	CON	0.32	0.73	0.41	**82.01**	98.59
	SF	0.80	1.19	0.89	**12.71**	13.92

The best recovery results of the three methods are shown in bold.

**Table 3:** No-reference evaluation indexes values performed on Fig. 3

Image	Metrics	Methods			
		Blurry	Blind Deconv	Wiener	CycleGAN
a	AG	3.11	4.54	2.79	**5.81**
	CON	13.85	33.95	14.38	**74.57**
	SF	5.23	7.77	5.31	**12.1**
b	AG	10.40	10.75	9.40	**11.06**
	CON	157.9	178.88	142.76	**202.73**
	SF	17.64	18.79	16.69	**20.01**
c	AG	2.89	3.93	3.05	**5.02**
	CON	14.25	28.15	20.75	**54.02**
	SF	5.30	7.45	6.32	**10.29**

The best results are shown in bold.

**Table 4:** No-reference evaluation indexes values performed on Fig. 4.

Methods	Metrics		
	AG	CON	SF
Blurry	2.41	8.08	4.01
CycleGAN	3.08	37.16	8.53

To visually and comprehensively demonstrate the deblurring effects of each model, three no-reference image quality evaluation metrics, Average gradient (AG)^46^, Contrast (CON)^47^, and Spatial frequency (SF)^48^, were also used to evaluate the results in Fig. 2 and Supplementary Fig. S1. AG is the average value of the image gradient. CON measures the contrast of the image by the gray difference between adjacent pixels and the pixel distribution probability. SF reflects the change rate of the image grayscale, which is used to measure the overall activity level of an image. The values of AG, CON, and SF are numbers greater than or equal to zero but have no upper limit, the larger the values, the clearer the image. Further details on the image quality evaluation metrics are presented in the “Methods” section. As shown in Table 2 and Supplementary Table S2, the CycleGAN model achieved the maximum values for the three metrics, which were closest to the ground truth, indicating that the CycleGAN model can effectively improve image sharpness and highlight image details. Conversely, traditional methods had poor performance on the image restoration, especially in cases involving complex blur.

### Results on the real dataset

To evaluate the deblurring capability of our model in real data, the model was trained and tested on the real dataset. The real dataset consists of unmatched clear and blurry SEM images obtained from experiments. Clear SEM images are obtained by SEM imaging of materials with good conductivity. The blurry images are obtained by SEM imaging of the above materials after introducing organic contamination. Figure 3 and Supplementary Fig. S2 show the SEM image deblurring results of various models on different samples. The materials shown in Fig. 3a–c were tungsten trioxide ($\mathrm WO_{3}$) and copper sulfide (CuS), respectively. Subjectively, compared with the traditional methods, the recovered images obtained by our method have clearer edges, better contrast, and richer details. Objectively, the recovered images were evaluated by the no-reference image quality evaluation metrics, and the results are shown in Table 3 and Supplementary Table S3. It can be seen that the recovered images obtained by the CycleGAN model achieve the maximum values for all metrics, consistent with the results obtained from the simulated dataset. These results indicate that the CycleGAN model used here has stable performance on images of different materials and can adapt to different degrees and types of blurriness, enhancing image detail information and clarity.

In addition to weakly conducting samples obtained by adding organic contaminants, we also verified the effectiveness of our method on SEM images of weakly conducting material that has not been trained by a network. Figure 4a shows the SEM image of silicon dioxide (${\text{SiO}}_{2}$) particles. Due to its intrinsic weak conductivity, the high-magnification SEM image of ${\text{SiO}}_{2}$ has low imaging quality which is not clear and the edges are blurred. After processing with the CycleGAN model, the image quality has significantly improved, and the particle edges are clearer (Fig. 4b). The rise in numerical values for multiple evaluation metrics further confirms this conclusion (Table 4). Therefore, our method can effectively improve the SEM imaging quality of weakly conductive materials.

### Edge Loss

As SEM images of micro-nano scale materials often exhibit rich edge details, an additional edge loss was incorporated when constructing the network. Here, the effects of the edge loss on the recovered images were investigated. As a crucial parameter, the value of edge loss weight $\gamma$ directly influences the quality of the generated images. If $\gamma$ is too small, the generator tends to produce artifacts in the output. Conversely, if $\gamma$ is too large, the generator prioritizes maintaining the input image, leading to a decrease in quality. The value of $\gamma$ in our model was determined through quantitative evaluation of synthetic data, as shown in Fig. 5a. As $\gamma$ increases from 0 to 20, the SSIM and PSNR values increase first and then decrease. Both reach their maximum values simultaneously when $\gamma$ is 10. Based on this, the value of $\gamma$ in our model was set as 10.

To further validate the effectiveness of the edge loss, the blurry SEM images were processed by the CycleGAN model with and without edge loss, as shown in Fig. 5b. Compared to the original image, both models enhanced the clarity and contrast of the images. However, the model without edge loss resulted in obvious artifacts on the edge of the material. The model with edge loss could maintain the edge details of the material, thus confirming the effectiveness of our edge loss. Furthermore, experiments were performed using the other operator as edge loss. Supplementary Fig. S3 and Table. S4 show that both Kirsch and Sobel operators can effectively restore the edge information of the image. The results demonstrated the validity of adding edge loss. Compared to multiple operators, the Sobel operator performs well and has low computational complexity, making it particularly suitable for our task.

## Conclusions

In summary, an unsupervised method based on CycleGAN was proposed to enhance the SEM imaging quality for weakly conductive samples. In the case of unknown blurry kernels and the absence of paired datasets, the proposed method effectively improves the quality of various blurry SEM images, including the restoration of image details, contrast, and improvement of clarity. The performance surpasses traditional methods significantly. In comparison to the reported CycleGAN architectures, we introduced an additional edge loss function tailored to material analysis needs, resulting in the removal of artifacts and restoring material contour details. As far as we know, this is the first application of unsupervised learning in improving SEM image quality. We believe that the work contributes to the expansion of artificial intelligence applications in materials science and has significant importance for material analysis.

## Methods

### Image quality metrics

AG is defined as follows:

1 $$\begin{aligned}{} & {} AG=\frac{1}{m\times n}\sum _{\mathrm {x=1}}^m\sum _{\mathrm {y=1}}^n\sqrt{\frac{\left( \left( f\left( x+1,y\right) -f\left( x,y\right) \right) ^2+\left( f\left( x,y+1\right) -f\left( x,y\right) \right) ^2\right) }{2}} \end{aligned}$$

where *f*(*x*, *y*) is pixel intensity of the image at (*x*, *y*), which is grayscale value in our work.

CON is defined as follows:

2 $$\begin{aligned} CON=\sum _{\delta }\delta (x,y)^{2}P_{\delta }(x,y) \end{aligned}$$

where $\delta$ is grayness difference between adjacent pixels, $P_\delta$ is the pixel distribution probability.

SF is defined as follows:

3 $$\begin{aligned} SF=\sqrt{RF^{2}+CF^{2}} \end{aligned}$$

where *RF* and *CF* are row frequency and column frequency respectively:

4 $$\begin{aligned}{} & {} RF=\sqrt{\frac{1}{m\times n}\sum _{x=1}^{m}\sum _{y=1}^{n}\left( f(x,y)-f(x,y-1)\right) ^2} \end{aligned}$$

5 $$\begin{aligned}{} & {} CF=\sqrt{\frac{1}{m\times n}\sum _{x=1}^{m}\sum _{y=1}^{n}\left( f(x,y)-f(x-1,y)\right) ^2} \end{aligned}$$

SSIM can be expressed as follows:

6 $$\begin{aligned}{} & {} SSIM(A, B, i)=\frac{\left( 2 \mu _A \mu _B+C 1\right) \left( 2 \sigma _{A B}+C 2\right) }{\left( \mu _A^2+\mu _B^2+C 1\right) +\left( \sigma _A^2+\sigma _B^2+C 2\right) } \end{aligned}$$

where $\mu$ and $\sigma$ are the mean and standard deviation of the images at pixel i over the 11 $\times$ 11 Gaussian filter, respectively. *C*1 and *C*2 are non-zero constants introduced to avoid the denominator from being 0. Usually, the *C*1 and *C*2 are much less than 1. We set the values of *C*1 and *C*2 as 0.0001 and 0.0004, respectively.

PSNR is defined as follows:

7 $$\begin{aligned}{} & {} PSNR=10\log _{10}\left( \frac{\max (I)^2}{{MSE}}\right) \end{aligned}$$

8 $$\begin{aligned}{} & {} {M S E}=\frac{1}{n} \sum _{i=1}^n\left( a_i-b_i\right) ^2 \end{aligned}$$

*max*(I) is the maximum pixel value, which is equal to 1 for normalized images. *MSE* is the mean squared error difference between the two images.

### Loss function

The adversarial loss ($L_{GAN}$) for generator *G* and the discriminator $D_B$ is specified as follows:

9 $$\begin{aligned} L_{G A N}\left( G, D_B, A, B\right) =\frac{1}{2} * \left[ \left( D_B(G(a))-1\right) ^2+\left( D_B(b)-1\right) ^2+D_B(G(a))^2\right] \end{aligned}$$

where *A* and *B* are unpaired blurred and clear images, *a* $\in$ *A*, *b* $\in$ *B*. Similarly, the $L_{GAN}$ for generator *F* and the discriminator $D_{A}$ is specifically as follows:

10 $$\begin{aligned}{} & {} L_{G A N}\left( F, D_A, A, B\right) = \frac{1}{2} *\left[ \left( D_A(F(b))-1\right) ^2+\left( D_A(a)-1\right) ^2+D_A(F(b))^2\right] \end{aligned}$$

The cycle consistency loss ($L_{cyle}$) is as follows:

11 $$\begin{aligned}{} & {} L_{cyle}(G, F, A, B)=(1-{SSIM}(F(G(a)), a)) +(1-{SSIM}(G(F(b)), b)) \end{aligned}$$

Mean-squared error function(MSE) was used as the identity loss ($L_{id}$) which was imposed on both generators *G* and *F*, as shown below:

12 $$\begin{aligned} L_{i d}(G, F, A, B)=MSE(a-F(a))+MSE(b-G(b)) \end{aligned}$$

The edge loss ($L_{edge}$) can be expressed as follow:

13 $$\begin{aligned}{} & {} L_{edge }(G, F, A, B)={M S E}\left( g* F(G(a))-g* F(a)\right) +{M S E}\left( g* G(F(b))-g* G(b)\right) \end{aligned}$$

14 $$\begin{aligned}{} & {} g=\sqrt{g_x^2+g_y^2} \end{aligned}$$

The Sobel operators in the *x* and *y* directions are:

15 $$\begin{aligned} {g_x=\begin{bmatrix} -1&{}0&{}1\\ -2&{}0&{}2\\ -1&{}0&{}1 \end{bmatrix}\quad g_y=\begin{bmatrix} -1&{}-2&{}-1\\ 0&{}0&{}0\\ 1&{}2&{}1 \end{bmatrix}} \end{aligned}$$

Our final loss is defined as the weighted sum of the above four losses:

16 $$\begin{aligned} L(G, F, A, B)=L_{G A N}+\lambda _1 L_{c y c l e}+\lambda _2 L_{i d}+\gamma L_{edge } \end{aligned}$$

where coefficients $\lambda _1$, $\lambda _2$, and $\gamma$ are the weights of cycle consistency loss, identity loss, and edge loss, respectively. The weight size determines the influence of different losses on the overall loss function. The values of $\lambda _1$, $\lambda _2$, and $\gamma$ were empirically determined as 10, 5, and 10, respectively.

### Experimental settings

The proposed model was trained using the TensorFlow framework on an NVIDIA GeForce RTX 3090. Based on the computer hardware used, each experimental model was trained for 50 epochs with a batch size of 1. Adam Optimizer was used to optimize the gradients with a learning rate of 0.0001. The image size was set to a fixed resolution of 256 $\times$ 256 pixels for input to the network during training. All the images were acquired on the Hitachi SU8010 SEM that was used with a 5 kV accelerating voltage.

Synthesis dataset: Three synthetic datasets were created. The first dataset only adds Gaussian blur. Gaussian blur is an image blurring filter that uses the Gaussian distribution to calculate the transformation of each pixel in the image. In two-dimensional space, it is defined as:

17 $$\begin{aligned} G(x,y)=\frac{1}{2\pi \sigma ^{2}}e^{-(x^{2}+y^{2})/2\sigma ^{2}} \end{aligned}$$

where $\sigma$ is the standard deviation of the function, which controls the radial range of the function. The second dataset adds a random concentration synthetic fog and Gaussian blur. The formation of a foggy image can be formulated as follows:

18 $$\begin{aligned} I(x, y)=f(x, y)^{*} e^{-{\beta }d}+A\left( 1-e^{-{\beta }d}\right) \end{aligned}$$

where *I*(*x*, *y*) and *f*(*x*, *y*) refer to the foggy and original image, respectively. $\beta$ is the scattering coefficient, the *d* is the depth of field, and the *A* controls the light intensity. The $\beta$ is randomly chosen in the range of [1.5- 2.5], *d* is 1, and the *A* is 3. The third dataset adds hybrid blur (Gaussian blur, motion blur, out-of-focus blur), and synthetic fog. The motion blur can be expressed as follows:

19 $$\begin{aligned} H(x,y)={\left\{ \begin{array}{ll}1/d&{}if\sqrt{x^2+y^2}\le d/2;\\ {} &{}y/x=tan\theta \\ 0&{}otherwise\end{array}\right. } \end{aligned}$$

where the $\theta$ is the motion blur angle and the *d* is the motion blur length. The $\theta$ = 0$\circ$ and d = 10 pixels. The out-of-focus blur caused by a system with a circular aperture can be modeled as a uniform disk with a radius *r*:

20 $$\begin{aligned} K(x, y)=\left\{ \begin{array}{ll} 0 &{} \sqrt{x^{2}+y^{2}}>r \\ \frac{1}{\pi r^{2}} &{} \sqrt{x^{2}+y^{2}} \le r \end{array}\right. \end{aligned}$$

where the *r* is 5. The parameters of the applied Gaussian blur for all datasets were the kernel size and standard deviation, which were set as 7$\times$7 and 1, respectively. After data expansion, we obtained 2550 pairs of 256$\times$256 images, 10% of which were used for testing. During training, blurry and clear data sets were randomly shuffled to achieve unpaired data training.

Real dataset: We deliberately contaminated the samples to obtain blurry SEM images of weakly conductive samples, and collected SEM images of normal samples as clear images. After data expansion, we obtained 1550 pairs of 256 $\times$ 256 pixel size images, of which 10% were used for testing.

### Supplementary Information

Supplementary Information.

## Data Availability

The data used in this study are available upon request to the corresponding author.
