# Supplementary material for: Enhancing scanning electron microscopy imaging quality of weakly conductive samples through unsupervised learning
Source: Sci Rep. 2024 Mar 18;14:6439. doi: 10.1038/s41598-024-57056-4 (PMC10948821; doi:10.1038/s41598-024-57056-4)
Supplement: Supplementary file 1 — Supplementary Information. [file 41598_2024_57056_MOESM1_ESM.pdf]

Supplementary Material

Enhancing Scanning Electron Microscopy Imaging Quality of Weakly Conductive Samples through Unsupervised Learning

Xin Gao[1,+], Tao Huang[1,+], Ping Tang[1], Jianglei Di[1], Liyun Zhong[1] ,and Weina Zhang[1,*]

[1]Key Laboratory of Photonic Technology for Integrated Sensing and Communication, Ministry of Education, Guangdong University of Technology, Guangzhou 510006, China
[*]corresponding.author@email.example
[+]these authors contributed equally to this work

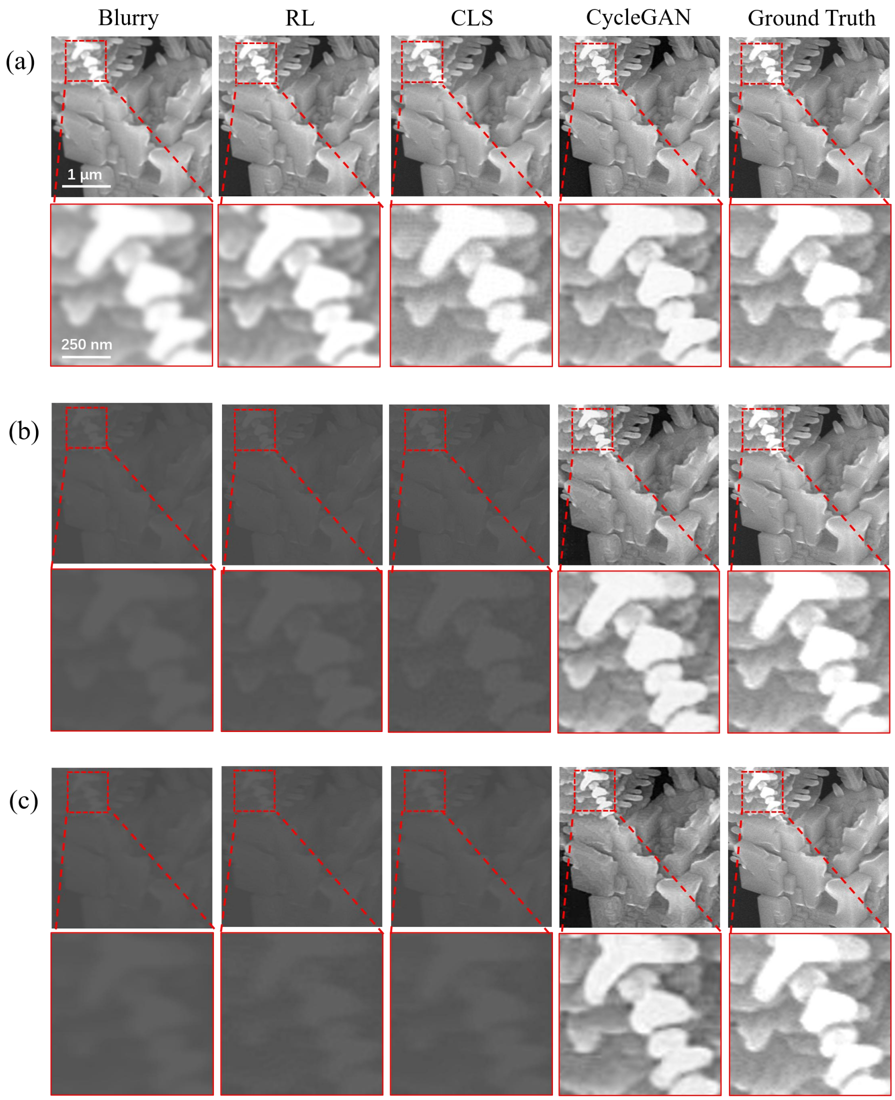

Supplementary Figure S1: Deblurring results of different models on simulated datasets. The material in the SEM images is iron chloride. (a) Deblurring results for data with Gaussian blur only. (b) Deblurring results for data with Gaussian blur and synthetic fog. (c) Deblurring results for data with synthetic fog and hybrid blur (Gaussian blur, motion blur, out-of-focus blur).

Dataset	Metrics	Methods			
		Blurry	RL	CLS	CycleGAN
A	SSIM	0.906	**0.929**	0.917	**0.929**
	PSNR (dB)	26.97	29.48	29.19	**29.77**
B	SSIM	0.861	0.859	0.812	**0.886**
	PSNR (dB)	25.99	26.33	24.69	**27.96**
C	SSIM	0.771	0.756	0.754	**0.798**
	PSNR (dB)	22.07	22.11	21.99	**24.27**

Supplementary Table S1: The average SSIM and PSNR of the deblurring results of the synthesized datasets, and the best results are shown in bold. A represents the data that only adding Gaussian blur. B represents the data that add Gaussian blur and the synthetic fog.

Image	Metrics	Methods				
		Blurry	RL	CLS	CycleGAN	Ground Truth
a	AG	3.11	4.96	3.62	**5.81**	7.37
	CON	13.85	33.57	18.23	**74.57**	98.59
	SF	5.23	8.12	5.99	**12.1**	13.92
b	AG	10.40	10.54	10.77	**11.06**	7.37
	CON	157.9	163.29	168.07	**202.73**	98.59
	SF	17.64	17.91	18.17	**20.01**	13.92
c	AG	2.89	4.31	4.23	**5.02**	7.37
	CON	14.25	29.62	28.06	**54.02**	98.59
	SF	5.30	7.62	7.44	**10.29**	13.92

Supplementary Table S2: No-reference evaluation indexes values performed on Supplementary Fig. S1. The best recovery results of the three methods are shown in bold.

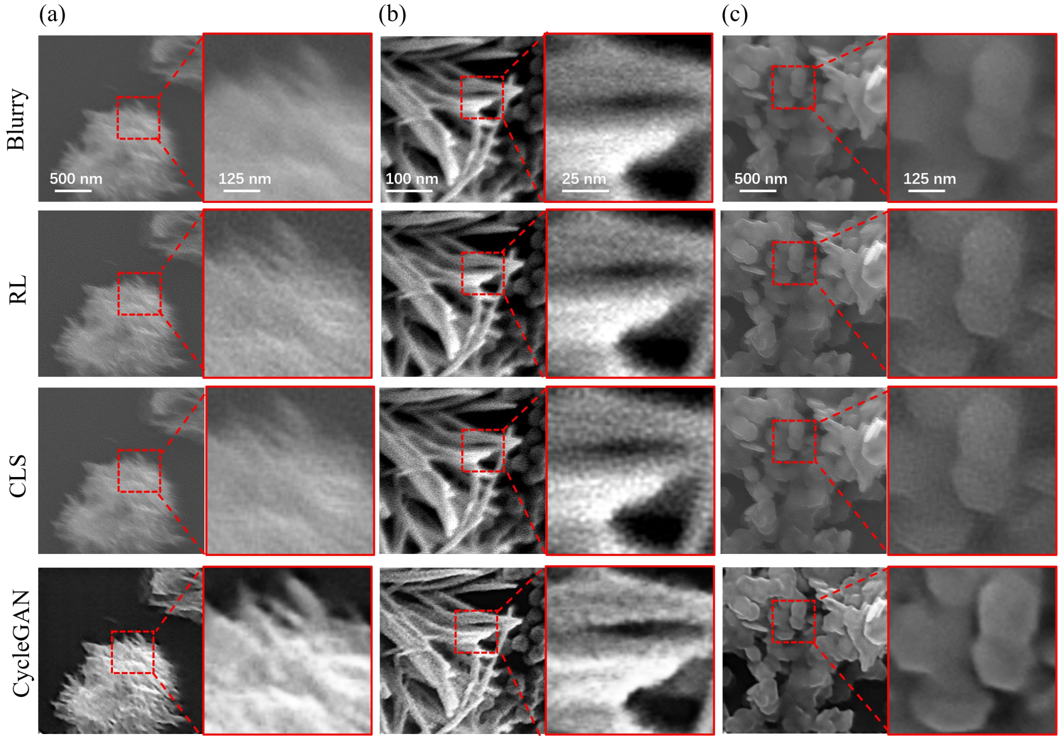

Supplementary Figure S2: Deblurring results of real data. In the red box are enlarged details in the corresponding dotted boxes. (a) Tungsten trioxide (WO_3) material.(b)Copper sulfide (CuS) material. (c)Tungsten trioxide (WO_3) with upconversion nanoparticles (UCNPs).

Image	Metrics	Methods			
		Blurry	RL	CLS	CycleGAN
a	AG	3.11	4.96	3.62	**5.81**
	CON	13.85	33.57	18.23	**74.57**
	SF	5.23	8.12	5.99	**12.1**
b	AG	10.40	10.54	10.77	**11.06**
	CON	157.9	163.29	168.07	**202.73**
	SF	17.64	17.91	18.17	**20.01**
c	AG	2.89	4.31	4.23	**5.02**
	CON	14.25	29.62	28.06	**54.02**
	SF	5.30	7.62	7.44	**10.29**

Supplementary Table S3: No-reference evaluation indexes values performed on Supplementary Fig. S2. The best results are shown in bold.

Subjectively, both Kirsch and Sobel operators can effectively restore the edge information of the image. From the perspective of no-reference evaluation metrics shown in Table S4, the using of Sobel operator is better to Kirsch operator for Fig. S3a and b, while it is opposite for Fig. S3c. Overall, the performance difference between these two operators is not significant, and they both perform quite well.

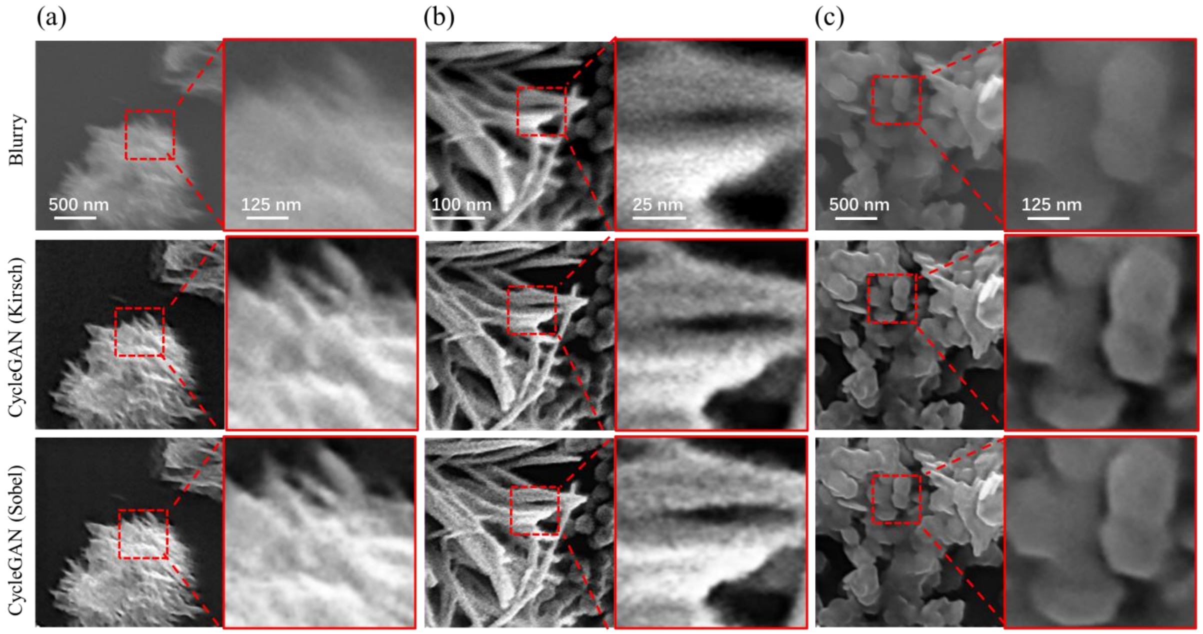

Supplementary Figure S3: Deblurring results of real data. The first row is the blurry image, the second row is the deblurred results using Kirsch operator, and the second row is the deblurred results using Sobel operator. (a)Tungsten trioxide (WO_3) material.(b)Copper sulfide (CuS) material. (c)Tungsten trioxide (WO_3) with upconversion nanoparticles (UCNPs).

Image	Metrics	Methods		
		Blurry	CycleGAN (Kirsch)	CycleGAN (Sobel)
a	AG	3.11	**6.093**	5.81
	CON	13.85	66.24	**74.57**
	SF	5.23	11.45	**12.1**
b	AG	10.40	10.309	**11.06**
	CON	157.9	173.63	**202.73**
	SF	17.64	18.54	**20.01**
c	AG	2.89	**5.37**	5.02
	CON	14.25	**59.27**	54.02
	SF	5.30	**10.81**	10.29

Supplementary Table S4: No-reference evaluation indexes values performed on Fig. S3. The best results are shown in bold.
